# ss3sim: An R Package for Fisheries Stock Assessment Simulation with Stock Synthesis

**DOI:** 10.1371/journal.pone.0092725

**Published:** 2014-04-03

**Authors:** Sean C. Anderson, Cole C. Monnahan, Kelli F. Johnson, Kotaro Ono, Juan L. Valero

**Affiliations:** 1 Earth to Ocean Research Group, Department of Biological Sciences, Simon Fraser University, Burnaby, British Columbia, Canada; 2 Quantitative Ecology and Resource Management, University of Washington, Seattle, Washington, United States of America; 3 School of Aquatic and Fishery Sciences, University of Washington, Seattle, Washington, United States of America; 4 Center for the Advancement of Population Assessment Methodology, La Jolla, California, United States of America; Institut Maurice-Lamontagne, Canada

## Abstract

Simulation testing is an important approach to evaluating fishery stock assessment methods. In the last decade, the fisheries stock assessment modeling framework Stock Synthesis (SS3) has become widely used around the world. However, there lacks a generalized and scriptable framework for SS3 simulation testing. Here, we introduce **ss3sim**, an R package that facilitates reproducible, flexible, and rapid end-to-end simulation testing with SS3. **ss3sim** requires an existing SS3 model configuration along with plain-text control files describing alternative population dynamics, fishery properties, sampling scenarios, and assessment approaches. **ss3sim** then generates an underlying ‘truth’ from a specified operating model, samples from that truth, modifies and runs an estimation model, and synthesizes the results. The simulations can be run in parallel, reducing runtime, and the source code is free to be modified under an open-source MIT license. **ss3sim** is designed to explore structural differences between the underlying truth and assumptions of an estimation model, or between multiple estimation model configurations. For example, **ss3sim** can be used to answer questions about model misspecification, retrospective patterns, and the relative importance of different types of fisheries data. We demonstrate the software with an example, discuss how **ss3sim** complements other simulation software, and outline specific research questions that **ss3sim** could address.

## Introduction

Fisheries stock assessment models are an invaluable tool for providing scientific advice regarding stock status, historical productivity, and changes in stock composition as well as evaluating the impact of alternative management actions on fishery resources [1], [2]. Although a variety of stock assessment approaches are available, it is often not straightforward to select among competing alternatives that may lead to different conclusions about stock status and associated scientific advice to management.

Simulation testing is a critical component to understanding the behavior of fishery stock assessment methods, particularly given the potential for model misspecification [2]–[6]. With simulation testing we can evaluate the precision and bias of alternative assessment approaches in a controlled environment where we know the true dynamics of hypothetical fisheries resources under exploitation. Recent simulation studies have been key to improving structural assumptions for dealing with, for example, time-varying natural mortality (*M*) [7]–[10], uncertainty in steepness of the stock-recruit relationship [11], and environmental variability [12], as well as determining the utility and influence on assessment outcomes of various fishery-dependent and -independent data sources [13]–[16].

There is a suite of tools available for conducting fishery stock assessments and Stock Synthesis (SS3, the third version of the software) is one, widely-used, modeling framework [17]. SS3 implements statistical age-structured population modeling using a wide range of minimally processed data [17], [18]. The generalized model structure of SS3 allows flexible scaling to a variety of data and life-history situations, from data-poor (e.g. [14], [19]) to data-rich (e.g. [20]). Owing in part to these advantages, SS3 has been used worldwide to formally assess 61 fishery stocks by 2012∶35 stocks in the US, 10 tuna/billfish stocks in three oceans, four European stocks, and 12 Australian stocks [17]. These assessments are conducted by both national agencies (e.g. NOAA in the United States of America, CSIRO in Australia) as well as regional fisheries management organizations (e.g. IATTC, ICCAT, IOTC in the Pacific, Atlantic and Indian oceans respectively). In addition to completed formal stock assessments, exploratory SS3 applications for many other stocks are underway [17].

Stock Synthesis is also commonly used as a framework for stock assessment simulation testing [7], [8], [11], [12], [16], [21]–[23], but there lacks a generalized structure for simulation testing with SS3. As a result, most stock assessment simulation-testing work using SS3 to date has relied on custom frameworks [6], [8], [9], [13], [14], [16], [21]–[24]. Although custom-designed modeling frameworks can be tailored to the specific needs of a particular stock assessment or simulation study, the use of a generalized framework allows other scientists to validate, reuse, and build upon previous work, thereby improving efficiency and resulting in more reliable outcomes.

The programming language R [25] is an ideal language in which to write such a generalized framework because (1) R has become the standard for statistical computing and visualization and (2) the R package **r4ss** [26] facilitates reading, processing, and plotting of SS3 model output. Here we introduce **ss3sim**, an R package that facilitates reproducible, flexible, and rapid end-to-end simulation testing with the widely-used SS3 framework. We begin by outlining the general structure of **ss3sim** and describing its functions, and then demonstrate the software with a simple example. We conclude by discussing how **ss3sim** complements other simulation testing software and by outlining some research questions that our freely accessible and general simulation-testing framework could address.

## The ss3sim Framework

### Design Goals of ss3sim

We designed **ss3sim** simulations to be reproducible, flexible, and rapid. *Reproducible*: **ss3sim** simulations are produced using R code, plain-text control files, and SS3 model configurations. **ss3sim** also allows for random seeds to be set when generating observation and process error. In combination, these features make simulations repeatable across computers and operating systems (Windows, OS X, and Linux). *Flexible*: **ss3sim** inherits the flexibility of SS3 and can therefore implement many available stock assessment configurations by either modifying existing SS3 model configurations or by modifying generic life-history model configurations that are built into **ss3sim** (Text S1). Furthermore, **ss3sim** summarizes the simulation output into plain-text comma-separated-value (.csv) files allowing the output to be processed in R or other statistical software. Finally, the **ss3sim** source code is written under an open-source MIT license and can be freely modified. *Rapid*: **ss3sim** relies on SS3, which uses AD Model Builder [27] – a rapid and robust non-linear optimization software [28] – as a back-end optimization platform. **ss3sim** also facilitates the deployment of simulations across multiple computers or computer cores (i.e. parallelization), thereby reducing runtime. By using the vetted SS3 framework with the tested **ss3sim** package, the time to develop and run a large-scale simulation study can be reduced substantially, allowing for more time to refine research questions and interpret results instead of spending it developing and testing custom simulation frameworks.

### The General Structure of an ss3sim Simulation

**Figure 1:**
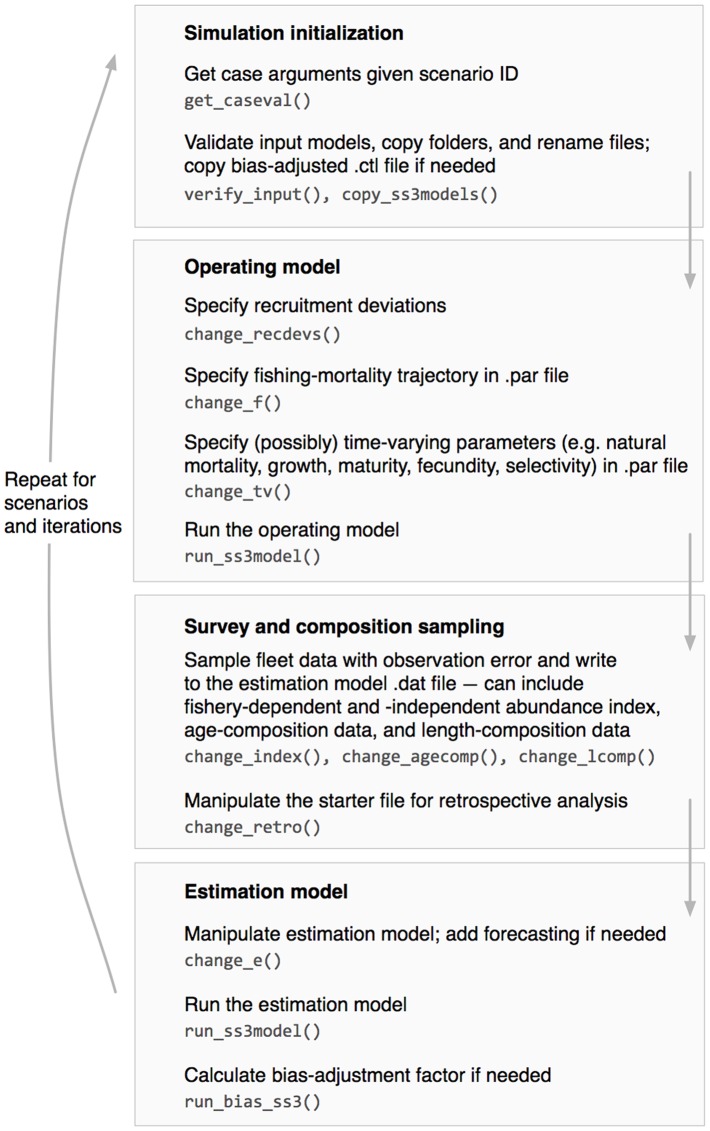
Flow diagram of the main steps in an ss3sim simulation carried out using run_ss3sim. Functions that are called internally are shown in a monospaced font.

**ss3sim** consists of both low-level functions that modify SS3 configuration files and high-level functions that combine these low-level functions into a complete simulation experiment (Figure 1, Table 1). In this paper we will focus on the structure and use of the high-level function run_ss3sim; however, the low-level functions can be used on their own as part of a customized simulation (see the R package vignette at http://cran.r-project.org/package=ss3sim).

**Table 1:** Main ss3sim functions and a description of their purpose.

Function name	Description
run_ss3sim	Main high-level function to run **ss3sim** simulations.
ss3sim_base	Underlying base simulation function. Can also be called directly.
change_rec_devs	Substitutes recruitment deviations.
change_f	Adds fishing mortality time series. (Case file and ID f)
change_tv	Adds time-varying features. For example, time-varying natural mortality, growth, or selectivity.(Any case file and ID, e.g. m, starting with “function_type; change_tv”)
change_index	Controls how the fishery and survey indices are sampled. (Case file index, case ID d)
change_agecomp	Controls how age composition data are sampled. (Case file agecomp, case ID d)
change_lcomp	Controls how length composition data are sampled. (Case file lcomp, case ID d)
change_retro	Controls the number of years to discard for a retrospective analysis. (Case file and ID r)
change_e	Controls which and how parameters are estimated. (Case file and ID e)
run_bias_ss3	Determines the level of adjustment to ensure mean-unbiased estimates of recruitment and biomass.
get_results_scenario	Extracts results for a single scenario.
get_results_all	Extracts results for a series of scenarios.

Simulations can be run through the run_ss3sim function, which then calls the change functions. Users can control what the change functions do through a series of plain-text case files. For example, the case ID d1 corresponds to the case files lcomp1, agecomp1, and index1, as described in the table. Users can also skip setting up case files and specify arguments to ss3sim_base directly, or use the change functions as part of their own simulation structure (see the vignette).

**Figure 2:**
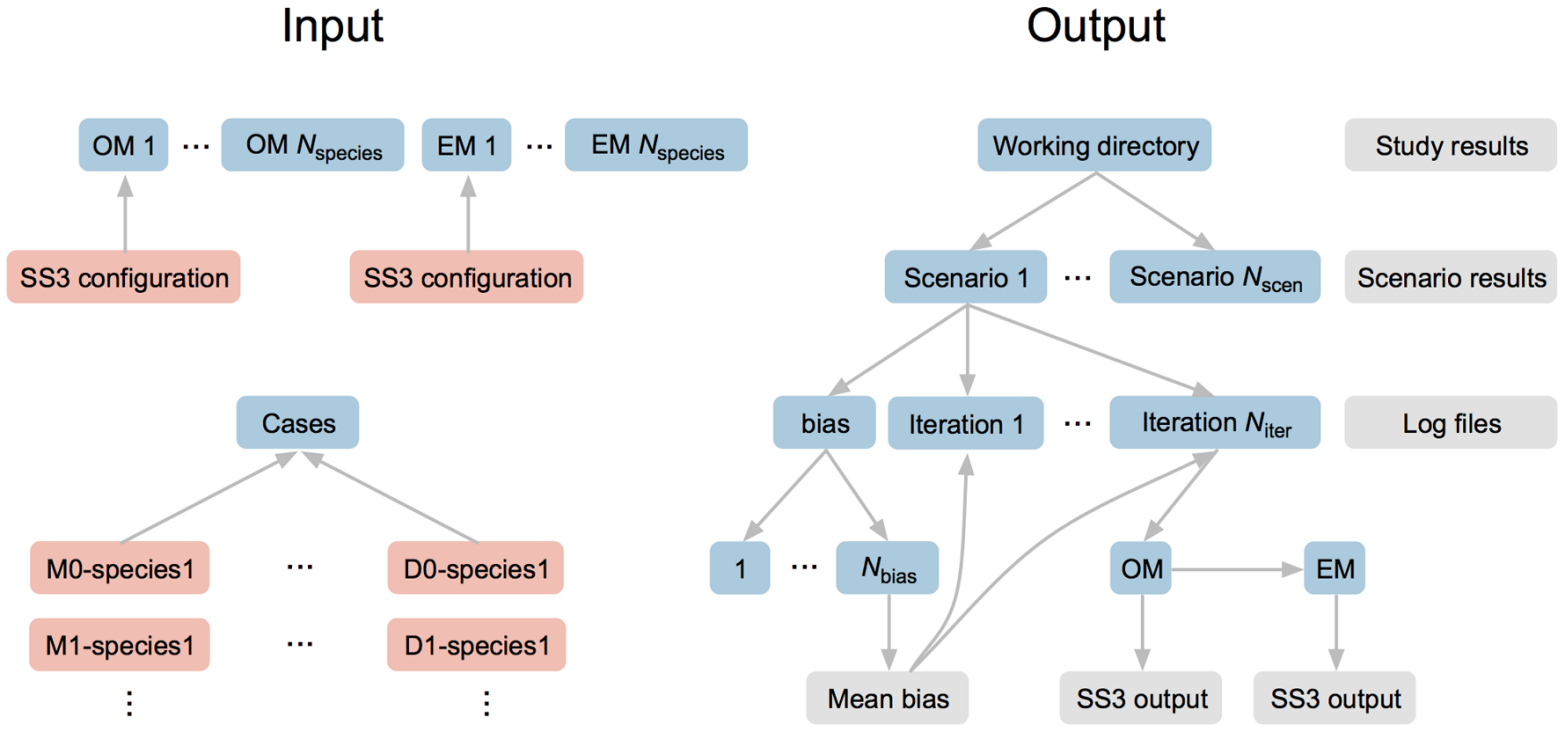
Illustration of input and output folder and file structure for an ss3sim simulation. Folders are shown in blue, input files in orange, and output files in grey. All input and output files are in plain text format. OM refers to operating model and EM to estimation model. Case files (orange files at bottom left) combine cases (e.g. M0 for a given natural mortality trajectory) with species or life-history OMs and EMs (e.g. cod-like or sardine-like). Alternatively, a user can skip setting up case files and specify the simulation cases directly in R code (see the vignette).

An **ss3sim** simulation requires three types of input: (1) a base SS3 model configuration describing the underlying true population dynamics, or operating model (OM); (2) a base SS3 model configuration to assess the observed data generated by the OM, also known as the estimation model or method (EM); and (3) a set of plain-text files (case files) describing alternative model configurations and deviations from these base models (e.g. different fishing mortality or *M* trajectories; Figure 2). We refer to each unique combination of OM, EM, and case files as a scenario. Scenarios are usually run for multiple iterations with unique process and observation error in each iteration. An **ss3sim** simulation therefore refers to the combination of all scenarios and iterations.

The run_ss3sim function works by modifying SS3 configuration files as specified in the case-file arguments (change functions), running the OM, sampling from the time-series of true population dynamics to generate an observed dataset (sample functions), running the EM to get maximum-likelihood estimates and standard errors of parameters and derived quantities, and synthesizing the output for easy data manipulation and visualization (get functions) (Figure 1).

### An Example Simulation with ss3sim

To demonstrate **ss3sim**, we will work through a simple example in which we examine the effect of (1) high vs. low precision of a fishery independent index of abundance and (2) fixing *M* at an assumed value vs. estimating *M*. All files to run this example are included in the package data, and a more detailed description is available in the accompanying vignette. **ss3sim** requires R version 3.0.0 or greater and SS3. In R, **ss3sim** can be installed and loaded with:

install.packages(“ss3sim”)

library(“ss3sim”)

Alternatively, the development version of **ss3sim** can be installed from https://github.com/ss3sim/ss3sim. You can read the documentation and vignette with:

?ss3sim

vignette(“ss3sim-vignette”)

### Setting Up the SS3 Model Configurations

**ss3sim** comes with built-in SS3 model configurations that represent three general life histories: cod-like (slow-growing and long-lived), flatfish-like (fast-growing and long-lived), and sardine-like (fast-growing and short-lived). These model configurations are based on North Sea cod (*Gadus morhua*; R. Methot, NMFS, NOAA; pers. comm.), yellowtail flounder (*Limanda ferruginea*; R. Methot, NMFS, NOAA; pers. comm.), and Pacific sardine (*Sardinops sagax caeruleus*) [29] (see the vignette). We recommend modifying these built-in model configurations to match a desired scenario, although it is possible to create a new **ss3sim** model by modifying an existing SS3 model configuration (see the vignette). We will base our example around the built-in cod-like model setup.

### Setting Up the Case Files

The high-level function run_ss3sim can run all simulation steps based on a specified scenario ID and a set of semicolon-delimited plain-text files that describe alternative cases (Figures 1 and 2). These case files contain argument values that are passed to the low-level **ss3sim** R functions (e.g. change_index, a function that controls how the fishery and survey indices are sampled; Table 1).

To use run_ss3sim, all case files must be named according to the type of case (e.g. E for estimation or F for fishing mortality), a numeric value representing the case number, and an alphanumeric identifier representing the species or stock (e.g. cod; Table 1, R package vignette). We combine these case IDs with hyphens to create scenario IDs. For example, one of our scenarios will have the scenario ID D1-E0-F0-M0-R0-cod. This scenario ID tells run_ss3sim to read the case files corresponding to the first data (D) case (i.e. index1-cod.txt, lcomp1-cod.txt, agecomp1-cod.txt), the zero case for estimation (E; i.e. E0-cod.txt), and so on.

To investigate the effect of different levels of precision of a fishery-independent index of abundance, we will manipulate the argument sds_obs that gets passed to the function change_index. In data case D0, we will specify the standard deviation of the index of abundance at 0.1 and in case D1 we will increase the standard deviation to 0.4. We can do this by including the line: sds_obs; list(0.1) in the file index0-cod.txt and the line: sds_obs; list(0.4) in the file index1-cod.txt. We will also set up a base-case file describing fishing mortality (F0-cod.txt), a file describing a stationary *M* trajectory (M0-cod.txt), and specify that we do not want to run a retrospective analysis in the file R0-cod.txt. We will set up the file E0-cod.txt to fix *M* at the true value and not estimate it, and case E1-cod.txt to estimate a stationary, time-invariant *M* (see the vignette).

All of these text files are available in the package data in the folder inst/extdata/eg-cases/. As an example, here is what the complete index0-cod.txt file looks like:

fleets; 2

years; list(seq(1974, 2012, by = 2))

sds_obs; list(0.1)

fleets, years, and sds_obs refer to the arguments in the function change_index and users can read the help for this function with ?change_index in R.

### Validating the Simulation Setup

Before running and interpreting the results of a simulation, it is important to validate the testing framework at several levels. First, it is important to test that the functions that manipulate model configurations (i.e. the change functions) are set up properly. **ss3sim** comes with prepackaged models that have been tested extensively with the change functions, as well as documented R functions that include examples and unit tests. We describe strategies for testing the change functions on new SS3 model setups in the R package vignette.

Second, the components of the simulation framework must work together as expected (integration tests [30]). One approach to testing for such issues is to run simulation tests with similar OM and EM setups and relatively low process and observation error [2]. **ss3sim** makes this form of validation simple by allowing users to specify levels of process and observation error (see the vignette). Assuming that the user specifies sufficient error to avoid numerical instability, this approach can reveal issues that would otherwise be obscured by noise.

Finally, it is important to validate that the model-fitting algorithms converged to global maxima. **ss3sim** retains all SS3 model output for future examination, as well as performance diagnostics such as maximum gradient, whether or not the covariance matrix was successfully calculated, run time, and the number of parameters stuck on bounds. These metrics, in combination with visual checks, are useful to determine if the results of a study are robust and meaningful.

### Running the Simulations

Since we have already validated the cod-like model setup (see the vignette), we can now run our example simulation scenario. To start, we will locate three sets of folders within the package data: the folder with the OM, the folder with the EM, and the folder with the plain-text case files:

d <- system.file(“extdata”, package = “ss3sim”)

om <- paste0(d, “/models/cod-om”)

em <- paste0(d, “/models/cod-em”)

case_folder <- paste0(d, “/eg-cases”)

We can then run the simulation with one call to the run_ss3sim function. We will set bias_adjust = TRUE to enable a procedure that aims to produce mean-unbiased estimates of recruitment and biomass despite log-normal recruitment deviations [31]. We can run 100 iterations of the simulation scenarios with the following code:

run_ss3sim(iterations = 1∶100, scenarios = 

c(“D0-E0-F0-M0-R0-cod”, “D1-E0-F0-M0-R0-cod”,

“D0-E1-F0-M0-R0-cod”, “D1-E1-F0-M0-R0-cod”),

case_folder = case_folder, om_dir = om,

em_dir = em, bias_adjust = TRUE)

This produces a folder structure in our working directory containing all of the SS3 output files (Figure 2). We can then collect the output with one function call:

get_results_all()

This command creates two files in our working directory: ss3sim_scalars.csv and ss3sim_ts.csv, which contain scalar output estimates (model parameters and derived quantities such as steepness and maximum sustainable yield) and time-series estimates (e.g. recruitment and biomass for each year). These estimates come from the report files produced from each run of SS3 as extracted by the **r4ss** R package. The.csv files contain separate columns for OM and EM values, making it simple to calculate error metrics, such as relative or absolute error. In addition to parameter estimates, the.csv files contain performance metrics, which in combination can be used to gauge model performance and convergence. These results are organized into “long” data format, with columns for scenario and iteration, facilitating quick analysis and plotting using common R packages such as **ggplot2** [32].

**Figure 3:**
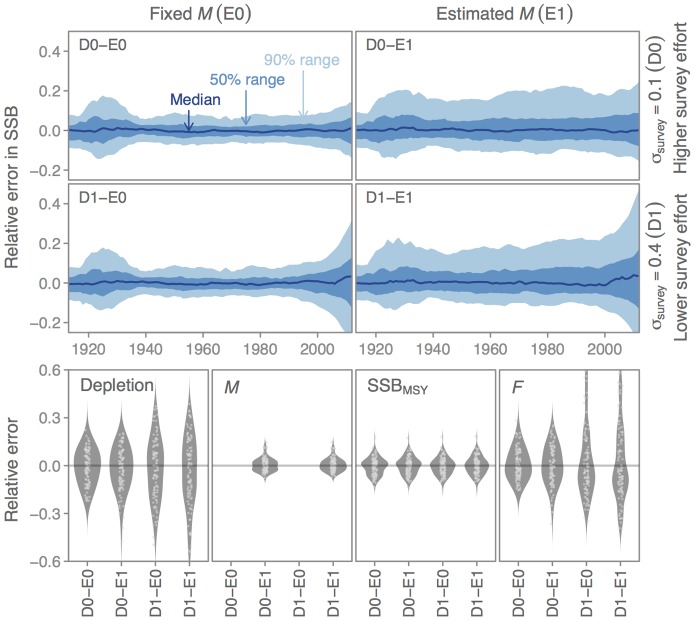
Example output from an ss3sim simulation. We ran a crossed simulation in which we considered (1) the effect of fixing natural mortality (*M*) at its true value (0.2; case E0) or estimating *M* (case E1) and (2) the effect of high survey effort (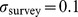; case D0) or low survey effort (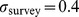; case D1). Upper panels (blue) show time series of relative error in spawning stock biomass (SSB). Lower panels (grey) show the distribution of relative error across four scalar variables: depletion (the ratio of terminal year spawning biomass to unfished spawning biomass), *M*, SSB at maximum sustainable yield (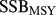), and fishing mortality (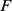) in the terminal year. We show the values across simulation iterations with dots and the distributions with beanplots (kernel density smoothers).

For the example simulation, the relative error in spawning stock biomass over time is, as expected, smaller when the true value of *M* is specified rather than estimated (Figure 3, top panels E0 vs. E1). Furthermore, lower precision in the research survey index of abundance results in greater relative error in spawning stock biomass in recent years (Figure 3, top panels D0 vs. D1), and greater relative error in terminal-year depletion (the ratio of terminal year spawning biomass to unfished spawning biomass) and fishing mortality, but not in spawning stock biomass at maximum sustainable yield, or *M* (Figure 3, lower panels).

### How ss3sim Complements other Simulation Software

The general purpose of **ss3sim** is to explore model behaviour and performance across combinations of EM configurations and alternative dynamics of fisheries resources under exploitation specified by the OM. In particular, **ss3sim** provides a suite of functions for dynamically creating structural differences in both OMs and EMs. This expedites testing the properties of alternative stock assessment model configurations, whether the differences are between OMs and EMs [10], or between multiple versions of EMs [15]. However, **ss3sim** is less suited for quickly exploring new SS3 model setups, which may rely on SS3 configurations not yet converted to work with the **ss3sim** package functions. Although it is possible to adapt arbitrary SS3 models to work with **ss3sim** (R package vignette), other software frameworks may provide better alternatives, depending on the goal of the simulation study.

One alternative software framework is Fisheries Libraries in R (FLR) [33] – a collection of open-source R packages developed specifically for evaluating fisheries management strategies through simulation. Compared to **ss3sim**, FLR is designed to explore broader questions regarding management strategies with flexible biological, economic, and management components [34]. Thus, it is not specifically designed to explore the impact of structural differences within OMs and EMs.

Another alternative stock assessment simulation testing framework is Fishery Simulation (FS, http://fisherysimulation.codeplex.com). FS is primarily a file management tool adapted to aid in simulation testing. FS can work with stock assessment models besides SS3, make simple changes to input text files, generate simple random process errors (using a built-in random number generator) and observation errors (using the SS3 bootstrap option), run simulations in parallel, and collect results from output files. Thus, FS is closer to **ss3sim** in its scope than FLR in that it specifically focuses on the performance of stock assessment models. FS differs from **ss3sim** mainly in that it uses user-specified text manipulation commands (e.g. change line 50 from 0 to 1) to alter model configurations rather than the approach of **ss3sim**, which uses modular functions tailored to specific purposes (e.g. add a particular time-varying mortality trajectory to an arbitrary OM). FS works well for testing arbitrary assessment models and model configurations because it does not rely on pre-built manipulation functions [7], [11], [35]. In contrast, FS cannot make complicated structural changes to a model setup (e.g. adding time-varying parameters or changing the survey years), limiting its ability to induce and test structural differences between OMs and EMs. In addition, the current version of FS is not an end-to-end package – additional code is necessary to incorporate arbitrary process and observation error in simulation testing. Finally, although FS is also open-source, it requires the Microsoft.NET framework and is therefore only compatible with the Windows operating system.

## Research Opportunities with ss3sim

The **ss3sim** package has been used so far to evaluate alternative assessment approaches when *M* is thought to vary across time [10], the effect of various qualities and quantities of length- and age-composition data on the bias and accuracy of assessment model estimates [15], and the causes of retrospective patterns in stock assessment model estimates. Along with those studies, **ss3sim** makes many relevant research opportunities easily approachable. Below we outline some examples.

### Time-varying Model Misspecification

Ecological processes can vary through time in response to, for example, changes to fishing behaviour [2], regime shifts [36], or climate change [37]. However, parameters such as *M*, catchability, and selectivity are commonly assumed to be time invariant and the consequences of these assumptions when facing true temporal changes has been a long-standing discussion in fisheries science [24], [38], [39]. Furthermore, although studies have tried to isolate the effects of single time-varying parameter, such as *M* [7]–[10], few have considered the effects of multiple time-varying parameters and their potential interaction. **ss3sim** can easily turn parameter estimation on and off as well as add time-varying dynamics to the OM, making it an ideal candidate for assessing the effects of multiple time-varying parameters.

### Patterns in Recruitment Deviations

Typically, estimation methods assume independent log-normally-distributed recruitment deviations around a spawning stock recruitment function. However, recruitment deviations are frequently auto-correlated and their variability can change through time [40], [41]. **ss3sim** facilitates exploring the effect of different recruitment deviation structures on model performance by allowing the user to directly specify any vector of deviations.

### Retrospective Patterns

Retrospective patterns, in which model estimates are systematically biased with each additional year of data, are a major problem in stock assessment science [42], [43]. Key questions include what causes retrospective patterns and which assessment approaches reduce them [43]. **ss3sim** can run retrospective analyses as part of any simulation by adding a single argument: the number of retrospective years to investigate.

## Conclusions

The increasing complexity of modern integrated stock assessment models and expanding computing power allows for the inclusion of multiple sources of data and estimation of increasingly complex processes [18]. However, it is difficult to determine under which conditions these processes can be reliably estimated based on diagnostics such as residual patterns [18]. Simulation testing is an important tool because it provides an opportunity to explore model performance under specified conditions and develop a further understanding of a model’s abilities.

We anticipate that **ss3sim** will facilitate the development of reliable assessment methods, applicable to age-structured stock assessment frameworks in general, that meet the requirements and assessment demands of many regional fisheries management organizations and national assessment agencies. For example, Johnson et al. [10] used **ss3sim** to develop guidelines for how to model natural mortality (when it is suspected of being time varying but age invariant) across life histories and fishing patterns. As another example, Ono et al. [15] used **ss3sim** to identify the most informative combination of quantity, quality, and timing of data, depending on life history and stock-assessment-derived metrics of interest. General guidelines such as these, combined with simulations testing specific model configurations used by assessment agencies, are an important part of developing reliable assessment methods to provide sound scientific advice to fisheries management [6], [44].

Custom-tailored simulation-testing software packages are an increasingly common tool in fisheries science, but their value would be extended if shared formally with the broader community. Published, open-source simulation frameworks, such as the initial release of **ss3sim** described here, allow other scientists to validate, reuse, and improve the software. We therefore encourage authors to publish their simulation frameworks and develop them in a generalized format, where possible. We anticipate that users will both benefit from **ss3sim** in its current form and extend it for their own needs, potentially contributing to future versions.

## References

[1] Gulland JA (1983) Fish Stock Assessment: A Manual of Basic Methods. New York: Wiley. 223 p.

[2] Hilborn RW, Walters C (1992) Quantitative Fisheries Stock Assessment: Choice, Dynamics, and Uncertainty. New York: Chapman and Hall. 570 p.

[3] Hilborn R, Walters CJ (1987) A general model for simulation of stock and fleet dynamics in spatially heterogeneous fisheries. Can J Fish Aquat Sci 44: 1366–1369.

[4] Rosenberg AA, Restrepo VR (1994) Uncertainty and risk evaluation in stock assessment advice for U.S. marine fisheries. Can J Fish Aquat Sci 51: 2715–2720.

[5] Peterman RM (2004) Possible solutions to some challenges facing fisheries scientists and managers. ICES J Mar Sci 61: 1331–1343.

[6] Deroba JJ, Butterworth DS, Methot RD, De Oliveira JAA, Fernandez C, et al.. (2014) Simulation testing the robustness of stock assessment models to error: some results from the ICES strategic initiative on stock assessment methods. ICES J Mar Sci. In press. doi:10.1093/icesjms/fst237

[7] Lee HH, Maunder MN, Piner KR, Methot RD (2011) Estimating natural mortality within a fisheries stock assessment model: An evaluation using simulation analysis based on twelve stock assessments. Fish Res 109: 89–94.

[8] Jiao Y, Smith EP, O’Reilly R, Orth DJ (2012) Modelling non-stationary natural mortality in catch-at-age models. ICES J Mar Sci 69: 105–118.

[9] Deroba JJ, Schueller AM (2013) Performance of stock assessments with misspecified age- and time-varying natural mortality. Fish Res 146: 27–40.

[10] Johnson KF, Monnahan CC, McGilliard CR, Vert-pre KA, Anderson SC, et al.. (2014) Time-varying natural mortality in fisheries stock assessment models: Identifying a default approach. ICES J Mar Sci. In press. doi:10.1093/icesjms/fsu055

[11] Lee HH, Maunder MN, Piner KR, Methot RD (2012) Can steepness of the stock-recruitment relationship be estimated in fishery stock assessment models? Fish Res 125–126: 254–261.

[12] Schirripa MJ, Goodyear CP, Methot RM (2009) Testing different methods of incorporating climate data into the assessment of US West Coast sablefish. ICES J Mar Sci 66: 1605–1613.

[13] Magnusson A, Hilborn R (2007) What makes fisheries data informative? Fish Fish 8: 337–358.

[14] Wetzel CR, Punt AE (2011) Performance of a fisheries catch-at-age model (Stock Synthesis) in data-limited situations. Mar Freshwater Res 62: 927–936.

[15] Ono K, Licandeo R, Muradian ML, Cunningham CJ, Anderson SC, et al.. (2014) The importance of length and age composition data in statistical age-structured models for marine species. ICES J Mar Sci. In press. doi: 10.1093/icesjms/fsu007

[16] Yin Y, Sampson DB (2004) Bias and precision of estimates from an age-structured stock assessment program in relation to stock and data characteristics. N Am J Fish Manage 24: 865–879.

[17] Methot RD Jr, Wetzel CR (2013) Stock Synthesis: A biological and statistical framework for fish stock assessment and fishery management. Fish Res 142: 86–99.

[18] Maunder MN, Punt AE (2013) A review of integrated analysis in fisheries stock assessment. Fish Res 142: 61–74.

[19] Cope JM (2013) Implementing a statistical catch-at-age model (Stock Synthesis) as a tool for deriving overfishing limits in data-limited situations. Fish Res 142: 3–14.

[20] Haltuch MA, Ono K, Valero JL (2013) Status of the U.S. petrale sole resource in 2012. Technical report, Pacific Fishery Management Council.

[21] Helu SL, Sampson DB, Yin Y (2000) Application of statistical model selection criteria to the Stock Synthesis assessment program. Can J Fish Aquat Sci 57: 1784–1793.

[22] Crone PR, Valero JL (In review) Evaluation of length vs. age selectivity in stock assessments based on management criteria: It matters when fitting to length-composition data.

[23] Hurtado-Ferro F, Punt AE, Hill KT (2013) Use of multiple selectivity patterns as a proxy for spatial structure. Fish Res. In press. doi:10.1016/j.fishres.2013.10.001

[24] Wilberg MJ, Bence JR (2006) Performance of time-varying catchability estimators in statistical catch-at-age analysis. Can J Fish Aquat Sci 63: 2275–2285.

[25] R Core Team (2013) R: A Language and Environment for Statistical Computing. R Foundation for Statistical Computing, Vienna, Austria.

[26] Taylor I, Stewart I, Hicks A, Garrison T, Punt A, et al.. (2013) r4ss: R code for Stock Synthesis. R package version 1.20.

[27] Fournier DA, Skaug HJ, Ancheta J, Ianelli J, Magnusson A, et al (2012) AD Model Builder: Using automatic differentiation for statistical inference of highly parameterized complex nonlinear models. Optim Methods & Soft 27: 233–249.

[28] Bolker BM, Gardner B, Maunder M, Berg CW, Brooks M, et al (2013) Strategies for fitting nonlinear ecological models in R, AD Model Builder, and BUGS. Methods Ecol Evol 4: 501–512.

[29] Hill KT, Crone PR, Lo NCH, Demer DA, Zwolinski JP, et al.. (2012) Assessment of the Pacific sardine resource in 2012 for U.S. management in 2013. Technical report, Pacific Fishery Management Council, Portland, Oregon, United States of America.

[30] Wilson G, Aruliah DA, Brown CT, Chue Hong NP, Davis M, et al (2014) Best practices for scientific computing. PLOS Biol 12: e1001745. 24415924 10.1371/journal.pbio.1001745 PMC3886731

[31] Methot RD, Taylor IG (2011) Adjusting for bias due to variability of estimated recruitments in fishery assessment models. Can J Fish Aquat Sci 68: 1744–1760.

[32] Wickham H (2009) **ggplot2**: Elegant Graphics for Data Analysis. New York: Springer. 213 p.

[33] Kell LT, Mosqueira I, Grosjean P, Fromentin JM, Garcia D, et al (2007) FLR: An open-source framework for the evaluation and development of management strategies. ICES J Mar Sci 64: 640–646.

[34] Hillary R (2009) An introduction to FLR fisheries simulation tools. Aquat Living Resour 22: 225–232.

[35] Piner KR, Lee HH, Maunder MN, Methot RD (2011) A simulation-based method to determine model misspecification: Examples using natural mortality and population dynamics models. Mar Coast Fish 3: 336–343.

[36] Vert-pre KA, Amoroso RO, Jensen OP, Hilborn R (2013) Frequency and intensity of productivity regime shifts in marine fish stocks. Proc Natl Acad Sci USA 110: 1779–1784. 23322735 10.1073/pnas.1214879110 PMC3562848

[37] Walther GR, Post E, Convey P, Menzel A, Parmesan C, et al (2002) Ecological responses to recent climate change. Nature 416: 389–395. 11919621 10.1038/416389a

[38] Royama T (1992) Analytical Population Dynamics. London: Chapman and Hall. 371 p.

[39] Fu C, Mohn R, Fanning LP (2001) Why the Atlantic cod (*Gadus morhua*) stock off eastern Nova Scotia has not recovered. Can J Fish Aquat Sci 58: 1613–1623.

[40] Beamish RJ (1995) Climatic change and northern fish populations. Can. Spec. Publ. Fish. Aquat. Sci. No.121.

[41] Pyper BJ, Peterman RM (1998) Comparison of methods to account for autocorrelation in correlation analyses of fish data. Can J Fish Aquat Sci 55: 2127–2140.

[42] Mohn R (1999) The retrospective problem in sequential population analysis: An investigation using cod fishery and simulated data. ICES J Mar Sci 56: 473–488.

[43] Legault CM (2008) Report of the retrospective working group. NOAA NMFS Northeast Fisheries Science Center Reference Document 09–01. Northeast Fisheries Science Center Reference Document 09–01, NOAA NMFS, Woods Hole, Massachusetts, United States of America.

[44] Crone PR, Maunder MN, Valero JL, D MJ, Semmens BX (2013) Selectivity: Theory, estimation, and application in fishery stock assessment models. Workshop series report 1. Center for the Advancement of Population Assessment Methodology, La Jolla, California, United States of America.

